# Simultaneous amplicon analysis of multiple soil samples using MinION sequencing

**DOI:** 10.1016/j.mex.2021.101576

**Published:** 2021-11-09

**Authors:** Hiroyuki Kurokochi, Kazutoshi Yoshitake, Ryo Yonezawa, Shuichi Asakawa

**Affiliations:** Graduate School of Agricultural and Life Sciences, The University of Tokyo, Yayoi 1-1-1, Bunkyo-ku, Tokyo 113-8657, Japan

**Keywords:** MinION, Soil microbes, Amplicon analysis, Next-generation sequencing

## Abstract

The diversity and composition of soil microorganisms needs to be understood as they influence ecosystem processes. MinION is a relatively recent next-generation sequencer, which provides the advantage of sequencing long reads. In this study, two types of soil were prepared experimentally to investigate the possibility of simultaneously analyzing multiple environmental samples using MinION. The MinION sequencing of amplicons was adjusted by the different rounds of PCR performed. Soil fungi and bacteria were compared using ITS and 16S rRNA amplicons, respectively. For ITS, the number of reads available for MinION sequencing were simply increased by performing two PCRs and purification using Agencourt AMPure XP. However, the effect of performing PCR twice was not high for 16S rRNA. Therefore, performing PCR twice appears to be effective for analyzing ITS regions. Regarding the number of reads obtained using MinION sequencing, clustering the same sample was possible if a read of ∼2000 bases or more was obtained in 16S rRNA and ITS. Further, information on 80 samples was obtained by performing only one round of MinION sequencing. Thus, MinION sequencing can be used to analyze a large number of samples simultaneously, providing a strong tool for amplicon sequencing.

• Soil microbial composition before and after treatment was compared between 16S rRNA and ITS amplicons using MinION sequencing

• One PCR amplification and two PCR amplifications were also compared

• Information on 80 samples was obtained by performing only one round of MinION sequencing

## Graphical abstract

**Figure:**
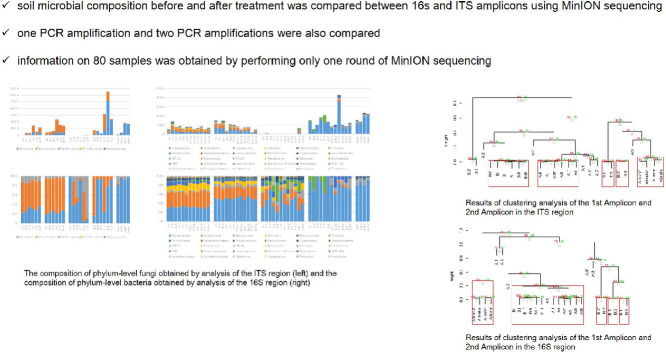

**Specifications Table**

**Table:** 

**Subject Area**	Biochemistry, Genetics and Molecular Biology
**More specific subject area**	Metagenome analysis, eDNA
**Method name**	Simultaneous amplicon analysis of multiple soil samples using MinION sequencing
**Name and reference of original method**	Nested PCR, tagged PCR
**Resource availability**	The original data of the metagenomic analysis used in this study is stored in the drive by the corresponding author (H.K.), so please contact H.K., if you would like to view it.

## Introduction

Forest ecosystems have developed a mechanism for nutrient recycling in internal circulation through interrelationships between plant-decomposers. Based on the mutual relationship between plants and decomposers, various organisms form communities using the organic matter produced as food and living resources [1]. (Takeda 1994). These decomposers are mainly soil microorganisms. As the diversity and composition of soil microorganisms influences ecosystem processes significantly [2,3], knowledge regarding soil microorganisms is an important factor in understanding forest ecosystems. Ongoing studies spanning many years have been undertaken to clarify the kind of soil microorganisms existing in the ecosystem, as an important theme related to soil microorganisms [4], [5], [6].

In recent years, next-generation sequencing has been widely used to understand the diversity of microorganisms [7]. Various next-generation sequencers are thus being used based on their respective merits [7,8]. MinION is a relatively recent next-generation sequencer, which provides the advantage of sequencing long reads and has been introduced at the laboratory level. Studies using MinION are also increasing steadily [9], [10], [11], [12]. Nygaard et al. [9] analyzed 12 samples of 16S amplicons with MinION. Peel et al. [10] reported that pollen collected from wild bees can be analyzed with MinION to reliably distinguish between species with high and low amounts of DNA in the sample.

Soil is a highly heterogeneous matrix [13], and sampling from multiple locations is essential to obtain the representative values ​​at a certain location. Samples from more points are needed for accurate analysis. Therefore, simultaneous analysis of multiple samples with a next-generation sequencer is important for conducting research on a limited budget.

In this study, we investigated the possibility of simultaneously analyzing multiple samples using MinION; two types of experimentally prepared soil were used, and MinION sequencing of amplicons adjusted by different rounds of PCR performed. Comparisons were made for soil microbial fauna (soil bacteria and fungi).

## Materials and methods

### Soil preparation for analysis

Brown forest soil collected from the red pine forest in Ina City, Nagano Prefecture, Japan, was air-dried for one year, mixed evenly, and sufficiently sieved through a sieve 2-mm pores. This soil was used as the “raw soil”. Further, Some of the raw soil was autoclaved at 121°C for 3 hours, and cooled at room temperature for 24 hours. This soil was used as “sterilized soil”.

Twelve plastic containers with lids were prepared. Six (I, II, III, IV, V, VI) of these were filled with 300 g of raw soil, and the remaining six (1, 2, 3, 4, 5, 6) were filled with 300 g of sterile soil.

At the beginning of the experiment, 2 g of soil was collected once from each of the 6 containers (I, II, III, 1, 2, 3) and stored in Unipack. From the remaining 6 containers (IV, V, VI, 4, 5, 6), 2 g of soil was collected twice from each container and stored in Unipack. Subsequently, several fallen leaves of *Euptelea polyandra* and several pill bugs were placed in each container, and stored in a cool and dark place (room temperature). In addition, some fallen leaves of *E. polyandra* were stored at -80°C. The inside of the container was humidified by adding 50 cc of distilled water, once a week. Two weeks after the start of the experiment, a total of 18 soil samples were collected in the same manner as described at the beginning of the experiment and were stored at -80°C.

### DNA extraction from soil samples

DNA was extracted from a total of 36 soil samples and 2 fallen leaves of E. polyandra using ISOIL (Nippon Gene Co., Ltd., Tokyo, Japan). The average amount of the soil samples used for DNA extraction was 0.30 g (s.d., 0.01 g), and that of*E. polyandra* samples was 0.04 g and 0.06 g. The concentration of each extracted DNA sample was measured using a Qubit2.0 Fluorometer (Thermo Fisher Scientific, Waltham, MA, USA).

### PCR amplification of extracted DNA

Amplicons of rDNA internal transcribed spacers (ITS) for soil fungal analysis and 16S rRNA (16S) for soil bacterial analysis were obtained as follows.

First, the amplicon obtained by PCR using the extracted DNA was defined as the 1st Amplicon. Furthermore, the amplicon obtained by PCR with a solution obtained by diluting the 1st Amplicon to 1/100 was defined as the 2nd Amplicon. MightyAmp DNA polymerase Ver. 3 (Takara Bio Inc., Shiga, Japan) was used for all PCR amplifications. The PCR solution was prepared by adding 12.5 μl of 2 × MightyAmp Buffer Ver.3, the forward and reverse primers to a final concentration of 0.3 μM each, 0.5 μl of MightyAmp DNA Polymerase Ver.3, 1 μl of extracted DNA, and 25 μl of sterile 1 μl of extracted DNA, and adjust the total volume to 25 μl with sterile water. The PCR conditions were as follows: treatment at 98°C for 2 minutes, followed by 35 cycles of 98°C-10 seconds, 60°C-15 seconds, and 68°C-2 minutes, and finally kept at 4°C.

For the 1st Amplicon of 16S, we designed multiple primers (1^st^ tagged primers; Appendix 1) with different 24 bp tags (1^st^ tags) added to the 5′ ends of 27F (5′AGAGTTTGATCMTGGCTCAG-3′) [14] and 1492R (5′GGTTACCTTGTTACGACTT-3′) [15]. In total, 38 extracted DNA samples derived from 36 soil samples and two *E. polyandra* leaves were used as the templates DNA for the 1st Amplicon, and PCR amplification was performed using different 1^st^ tagged primers for each sample. To obtain the 2nd Amplicon for the 16S sequence, we designed multiple primers (2^nd^ tagged primers) with different 26 bp tags (2^nd^ tags) at the 5′ end of each 1^st^ tag used in the 1st Amplicon (Appendix 2). The 1st Amplicon derived from the DNA extracted from the soil in six containers (I, II, III, 1, 2, 3) and from *E. polyandra* leaves were used as the template DNA of the 2nd Amplicon, and PCR amplification was performed using different the 2^nd^ tagged primers for each sample.

To obtain the 1st Amplicon and 2nd Amplicon of ITS, the primers of ITS1F (5′-TCCGTAGGTGAACCTGCGG-3′) [16] and ITS4 (5′-TCCTCCGCTTATTGATATGC-3′) [17] were used to design tagged primers (Appendix 1&2) that can identify the sample in the same flow as that used for the 16S as described above. PCR amplification was performed using tagged primers. The samples used to prepare the 1st Amplicon and 2nd Amplicon in the ITS region were the DNA samples obtained from the soil in six containers (I, II, III, 1, 2, 3) and from *E. polyandra* leaves.

### Amplicon sequencing and data analysis

In total, of 80 amplicons were obtained, and purified using Agencourt AMPure XP (Beckman Coulter, CA, USA). The concentration of the amplicons after purification was measured using a Qubit 2.0 Fluorometer (Thermo Fisher Scientific), and a mixture of all samples was prepared using the same amount of DNA, and this was used as a library. The library was processed using the Ligation Sequencing Kit (SQR-LSK109, Oxford Nanopore Technologies) and was subjected to amplicon sequencing using MinION (Oxford Nanopore Technologies).

The FASTA file of the primers was created based on the sequence of the barcoded primer, and a BLAST (ver.2.6.0+) database was created using the makeblastdb program. Of the reads obtained from the nanopores, 1200–1700bp for the 16S and 600–900 bp for the ITS sequences were targeted and executed by specifying "-word_size 4" in blastn, and regions homologous to the primers were searched. Only hits that were aligned with 90% or more of the primer sequence were used, and each read was classified using the primer names of the top hits on the forward and reverse sides. Reads with the barcoded primers at both ends were extracted, and sequences outside the PCR primers were trimmed. The above analysis was performed with “nanopore∼split-barcode” pipeline in Portable Pipeline (v. 0.9c) (https://github.com/c2997108/OpenPortablePipeline). For the 16S analysis, the metagenomics ∼ silva_SSU+LSU pipeline in Portable Pipeline (v. 0.9c) was used to estimate the taxa most applicable to each read. The option "-t 0.995" was specified. To briefly describe the pipeline, we performed a homology search using blast on the merged database of small and large subunits of SILVA (ver. 132), extracted reads with a bitscore of 100 or more, and aggregated the hits with a bitscore of 99.5% or more from the top hit to estimate the taxa by the lowest common ancestor method. In the analysis of ITS sequences, the Portable Pipeline "metagenome ∼ use-genbank-fasta-as-reference" pipeline was used, and in August 2019, 503,331 ITS sequences downloaded from NCBI GenBank were used as a database. The analysis of ITS was also performed using the same thresholds as for 16S.

In this study, we treated the estimated taxa as operational taxonomic units (OTUs), and the taxa estimated in the 16S and ITS were designated as OTU_16S and OTU_ITS, respectively. The number of OTUs and the index of diversity for each sample were calculated using R. Microbial composition was represented by Non-metric Multidimensional Scaling (NMDS) using the vegan package [18] and was clustered using OTUs with more than one confirmed read using the pvclust package [18].

## Results and discussion

### Concentrations of extracted DNA and PCR products

In this study, a total of 38 samples of extracted DNA were obtained from 36 soil samples and 2 *E. polyandra* fallen leaves. The concentration of each extracted DNA is shown in Appendix 3. Further, except for some samples, the sterile soil at the start of the experiment contained almost no DNA.

We obtained the 1st and 2nd Amplicon of 16S and ITS using 28 extracted DNAs: 3 extracted DNAs from sterilized soil before and after treatment, 3 extracted DNAs from raw soil before and after treatment, and 2 extracted DNAs from fallen leaves. The DNA concentrations of the 1st Amplicon and 2nd Amplicon of the 16S averaged 0.0935 μg/μl and 0.0968 μg/μl, respectively, and there was no significant difference in the concentration of PCR products depending on the number of PCR rounds performed. Further, the average DNA concentrations of the 1st Amplicon and 2nd Amplicon PCR of the ITS were 0.0081 μg/μl and 0.0896 μg/μl, and were significantly higher in the 2nd Amplicon. The amplification efficiency of the primers used at this time was different between the 16S and the ITS sequences, and the yield of PCR products could be increased by performing two PCR amplifications for ITS.

### The amount of DNA in each sample used for amplicon sequencing

A total of 80 samples, 52 for the 1st Amplicon and 28 for the 2nd Amplicon, were mixed. The amount of DNA provided by each sample was 0.49 ng (s.d., 0.102 ng). Sequencing was performed using MinION, and reads that met the set conditions were selected. Overall, 482965 reads were obtained from all 28 samples for analyzing the ITS sequences, of which 461484 reads were determined to be Fungi, spanning 3393 OTU_ITS. For the 16S analysis samples, 229718 reads were obtained from all 52 samples, of which 229502 reads spanned 6248 OTU_16S. Of the OTU_ITS and OTU_16S judged to be Fungi and Bacteria, two or more reads were 1774 OTU_ITS (total 459865 reads) and 3630 OTU_16S (total 226883 reads), which were used in the following analysis. The classification of each sample at the phylum level is shown in Appendix 4.

### Comparison of the 1st Amplicon and 2nd Amplicon

Comparing the number of reads obtained per amount of DNA, there was no significant difference between the 1st Amplicon (mean 10374 reads/ng) and 2nd Amplicon (mean 10807 reads/ng) for the entire 16S sample. On the contrary, for the entire ITS sample, the former had 11520 reads/ng, whereas the latter had 59003 reads/ng, showing a significant increase in the number of reads.

When comparing each treatment group, the values ​​of the Number of sequences obtained from MinION sequencing that matches the conditions set (N), Number of OTUs belonging to Fungi and containing two or more reads (OTU_ITS), Number of sequences included in OTU_ITS (Ns), and Ns in the 1 ng amplicons tested (Ns/ng) were all significantly increased in the 2nd Amplicon for ITS (Table 1). Nevertheless, there was no significant difference in the diversity index among any of the treatment groups (Table 1). Regarding the 16S sequences, there were no differences between the 1st Amplicon and 2nd Amplicon in raw soil, but a significant difference was observed when sterile soil was used (Table 2). On contrary, regarding the number of dominant species, there was no difference between the results of the 1st Amplicon and 2nd Amplicon (Table 2).

**Table 1:** Comparison of the 1st Amplicon and 2nd Amplicon in ITS

Sample collection	Sample	1^st^	2^nd^	1^st^	2^nd^	1^st^	2^nd^	1^st^	2^nd^	1^st^	2^nd^	1^st^	2^nd^
time	No.												
A		N *		OTU *		Ns *		Ns/ng *		S		R	
	I	113	32681	26	543	111	31956	234.7	77839.7	8.83	16.08	2.5	2.9
	II	8276	13727	307	360	8004	13227	21385.0	32211.7	14.30	12.78	2.5	2.4
	III	6679	26872	258	543	6544	26252	15301.3	63057.6	17.80	17.08	2.9	3.0
		N *		OTU *		Ns *		Ns/ng *		S		R	
	1	11	10026	6	35	10	2969	25.5	25318.2	4.47	1.49	2.0	1.1
	2	13	6448	10	37	12	2337	36.1	11611.7	9.52	4.25	6.0	1.6
	3	26	1969	7	42	12	945	74.1	4151.4	6.45	6.42	4.0	2.9
B		N *		OTU *		Ns *		Ns/ng *		S		R	
	I	6205	33850	197	426	6097	33159	12535.4	70301.1	15.28	14.89	3.0	3.0
	II	7035	23444	212	343	6860	22660	15326.8	50094.0	16.24	16.37	3.7	3.7
	III	9702	21515	282	399	9493	21024	17248.0	44683.3	15.82	15.60	3.5	3.4
		N *		OTU *		Ns *		Ns/ng *		S		R	
	1	12186	49923	116	205	12084	49313	29758.2	84687.0	6.66	8.18	2.8	3.0
	2	7530	81257	110	309	7481	80525	20658.4	145621.9	4.75	8.40	1.5	2.3
	3	4518	42264	173	537	4413	40848	16193.5	71151.5	19.87	22.88	3.4	3.6
A		N *		OTU *		Ns *		Ns/ng *		S		R	
	Leaves	2454	36853	163	488	2320	35048	4433.6	72473.9	18.73	14.03	5.1	3.9
		5492	31896	258	497	5291	30870	8082.4	72846.9	15.17	14.48	4.4	4.1

I–III and 1–3 are the sample numbers of containers containing the raw soil and sterile soil, respectively.

Leaves: Fallen leaves of *Euptelea polyandra*

1st and 2nd: 1st Amplicon and 2nd Amplicon

A and B: At the beginning of the experiment and after 2 weeks

N: Number of sequences obtained from MinION sequencing that match the conditions set in this study

OTU: Number of operational taxonomic units (OTUs) belonging to fungi and containing two or more sequences

Ns: Number of sequences included in the OTUs

Ns/ng: Ns in the 1 ng amplicons tested

S: Shannon Diversity Index

R: Reciprocal of the dominance rate in the most dominant species

⁎ : indicates a significant difference between the values of the 1st Amplicon and 2nd Amplicon.

**Table 2:** Comparison of the 1st Amplicon and 2nd Amplicon in 16S

Sample collection	Sample	1^st^	2^nd^	1^st^	2^nd^	1^st^	2^nd^	1^st^	2^nd^	1^st^	2^nd^	1^st^	2^nd^
time	No.												
A		N		OTU		Ns		Ns/ng		S		R	
	I	2728	4631	532	727	2695	4572	4663	7953	156	163	9	10
	II	7224	3056	901	609	7139	3021	16722	9525	207	184	12	12
	III	4079	4189	686	690	4034	4134	11621	11352	176	178	10	11
		N *		OTU *		Ns		Ns/ng *		S*		R	
	1	120	15	77	15	110	15	430	28	61	15	11	15
	2	307	14	127	10	300	14	784	31	84	8	14	3
	3	59	23	42	14	55	16	129	69	38	13	14	8
B		N		OTU		Ns		Ns/ng		S		R	
	I	7672	1919	949	461	7592	1901	22732	4959	216	165	14	17
	II	5935	5345	846	817	5870	5286	10304	11962	206	206	12	14
	III	7480	5175	973	859	7405	5108	15983	11165	241	231	15	16
		N *		OTU *		Ns		Ns/ng		S		R	
	1	7509	9236	223	270	7499	9206	13457	13775	17	20	4	5
	2	2821	10421	95	170	2811	10386	6669	18827	12	14	5	6
	3	5053	6979	233	301	5030	6937	10593	13486	20	28	3	4
A		N *		OTU		Ns		Ns/ng		S		R	
	Leaves	8020	12089	1169	1381	7781	11652	19164	26082	326	330	28	32
		6690	11235	788	1003	6561	10929	11989	22094	146	159	9	10

The abbreviations in the table are the same as those in Table 1.

With the exception of the samples derived from sterile soil at the beginning of the experiment (A: 1-3), the microbial composition of the 1st and 2nd Amplicon of each sample was almost the same, with distinct clustering (Appendix 5, 6). It was also shown that there was little difference in the microbial composition within the same treatment. On the other hand, samples derived from sterile soil at the beginning of the experiment (A: 1-3) had large differences in both the microbial composition between samples and the microbial composition between the 1st and 2nd Amplicon of the same sample (Appendix 5, 6). In these samples, the microorganisms were almost completely killed by autoclave, and little DNA could be extracted (Appendix 3). As a result, the number of reads for each sample obtained by MinION sequencing was also small, which may have prevented clear clustering even though the microbial composition was originally assumed to be similar.

### 1st Amplicon analysis of 16S

Regarding the raw soil, the number of reads obtained from the sample at the beginning of the experiment was 2684–7224 (average 3880), and the number of reads obtained from the sample two weeks later was 1013–7672 (average 4401) (Table 3). From the results of NMDS and clustering, the microbial composition of the raw soil samples was very similar for each group (Appendix. 7). From these results, it was possible that the information on microbial composition could be obtained if 1000 or more reads were obtained in the analysis using MinION. The difference among the samples at the beginning of the experiment is less than the difference among the samples after 2 weeks (Appendix 7). This is expected due to the growth of microorganisms over time. In terms of microbial composition, samples from the same container were not necessarily classified into the closest cluster (Appendix. 7). This result is presumed to be due to the high heterogeneity of the soil and the variation in the container, even though the soil samples were randomly prepared.

**Table 3:** Comparison of the 16S regions in soil DNA obtained from raw and sterile soil, containing repeats in the same container

SampleNo.	Sample collection time											
	A	B	A	B	A	B	A	B	A	B	A	B
	N		OTU		Ns		Ns/ng		S		R	
I	2728	7672	550	985	2695	7592	4663.2	22731.9	160.5	220.3	9.2	14.5
II	7224	5935	932	863	7139	5870	16722.2	10303.8	210.6	208.2	11.9	12.4
III	4079	7480	698	1007	4034	7405	11621.1	15982.9	178.3	246.3	10.3	15.4
IV-1	4156	4173	671	704	4099	4134	10629.2	7727.8	152.0	180.1	8.9	13.1
IV-2	2684	2855	551	580	2641	2829	4880.0	4798.3	130.2	184.5	8.2	21.1
V-1	3487	3681	622	678	3448	3651	6064.3	6239.0	151.3	189.0	10.2	16.5
V-2	4325	4071	678	694	4276	4023	7208.3	7469.7	143.4	166.3	7.7	15.7
VI-1	2750	1013	533	343	2719	1003	6084.1	2651.8	120.2	147.9	7.8	14.3
VI-2	3483	2732	659	582	3428	2707	6510.3	4371.2	186.3	170.9	11.3	11.8
Mean	3879.6	4401.3	654.9	715.1	3831	4357.1	8264.7	9141.8	**159.2**	**190.4**	**9.5**	**15**
s.d.	1407.24	2232.33	121.03	211.04	1392.22	2208.25	3978.11	6420.73	28.43	30.09	1.5	2.77
	N		OTU		Ns		Ns/ng		S		R	
1	120	7509	77	229	110	7499	430.1	13457.0	60.7	16.6	11.0	4.1
2	307	2821	128	97	300	2811	784.2	6669.0	84.8	11.7	14.3	5.5
3	59	5053	42	237	55	5030	128.5	10593.3	37.9	19.7	13.8	3.1
4-1	35	4502	24	421	28	4452	79.2	9272.9	21.9	46.6	7.0	4.3
4-2	170	5154	64	455	164	5092	387.2	7524.1	40.0	41.7	10.3	4.1
5-1	50	21523	35	585	47	21396	108.0	25232.1	26.1	17.2	4.7	2.1
5-2	156	4970	94	443	146	4918	370.1	9466.7	64.2	46.6	7.0	3.8
6-1	3356	4285	154	448	3334	4235	5918.9	8709.3	36.8	35.1	6.7	2.6
6-2	45	6038	22	249	33	6021	69.9	9079.7	17.5	10.6	5.5	1.9
Mean	**477.6**	**6872.8**	**71.1**	**351.6**	**468.6**	**6828.2**	**919.6**	**11111.6**	43.3	27.3	8.9	3.5
s.d.	1082.9	5638.61	46.77	154.57	1078.02	5609.46	1889.07	5631.95	22.27	15.04	3.54	1.17
	N		OTU		Ns		Ns/ng		S		R	
Leaves	8020		1169		7781		19164		326		28	
	6690		788		6561		11989		146		9	

I∼VI-2 and 1∼6-2 are the sample numbers of containers containing raw soil and sterile soil, respectively.

Leaves: Fallen leaves of *Euptelea polyandra*

Sample collection time: A and B indicate the beginning of the experiment and after 2 weeks, respectively

N: Number of sequences obtained from MinION sequencing that match the conditions set in this study

OTU: Number of operational taxonomic units (OTUs) belonging to Fungi and containing two or more sequences

Ns: Number of sequences included in the OTUs

Ns/ng: Ns in the 1 ng amplicons tested

S: Shannon Diversity Index

R: Reciprocal of the dominance rate for the most dominant species

Mean: The bold and underlined values indicate that the samples at the beginning of the experiment are significantly smaller and larger, respectively.

Regarding the sterile soil, the number of reads obtained from the sample at the start of the experiment was 35–3356 (average 478), and the number of reads obtained from the sample two weeks later was 2821–21523 (average 6873) (Table 3). Based on the NMDS and clustering results, there was a relatively large difference in the microbial composition between samples of sterile soil origin at the start of the experiment (Appendix 7). Ideally, since these samples were treated identically, the microbial composition should have been similar. However, one of the reasons why this was not the case was because of the small number of reads that we were able to obtain with MinION sequencing. Only one of the nine samples had more than 1000 reads at the start of the experiment, whereas the others were less than 500 (Table 3). Only the 6-1 sample had 3356 reads (Table 3), but as that the other samples had few reads, this could be a result of contamination. On the contrary, the microbial composition after the experiment was relatively similar (Appendix. 7) possibly because the microbial groups that dominated there were similar after almost the same treatment in all the containers, indicating a reasonable result. The index on species diversity was also decreased compared to that the start of the experiment (Table 3), suggesting that the species adapted under the current treatment conditions were dominant.

## Conclusion

In this study, two types of soil were prepared, and the soil microbial composition before and after the treatment was compared between 16S and ITS amplicons using MinION sequencing. The results of one PCR amplification and two PCR amplifications were compared to examine whether the amplification efficiency of the target region could be improved.

For ITS, the yield of the target PCR product was increased and the number of reads available for MinION sequencing was increased simply by performing two PCRs and purifying using Agencourt AMPure XP. On the contrary, in the 16S region, the effect of performing PCR twice was not high. Therefore, this method of performing PCR twice seems to be an effective means for analyzing the ITS region.

Regarding the number of reads obtained by MinION sequencing, clustering the same sample was possible if ∼2000 or more reads were obtained in 16S (Appendix 6, Table 2). For ITS, there were many samples with 2000 reads or more, and clustering and organism identification was achieved as expected (Appendix 5). However, only about 100 reads were obtained from one of the raw soil samples at the beginning of the experiment (Table 1), but the clustering results were accurate (Appendix 5). However, the species diversity of that sample was smaller than that of the other samples (Table 1). Some studies use tens of thousands of reads per sample for analysis, and this was less than that Sun et al. [5]; regarding the soil used in this study, the microbial composition could be evaluated if there were 2000 or more reads in both the 16S and ITS sequences.

Further, the results of this study provided information on a total of 80 samples by performing only one MinION sequencing. Despite exceptions such as sterilized soil where the amount of microorganisms in the initial state is extremely small and sufficient amplicons cannot be obtained, MinION sequencing can be used to analyze a large number of samples simultaneously, and can be used in the future to analyze environmental DNA. MinION sequencing can thus be a strong tool for amplicon sequencing.

## Declaration of Competing Interest

The authors declare no conflict of interest.

## Appendix Supplementary materials

Image, application 1
