# Supplementary material for: Simultaneous amplicon analysis of multiple soil samples using MinION sequencing
Source: MethodsX. 2021 Nov 9;8:101576. doi: 10.1016/j.mex.2021.101576 (PMC8720897; doi:10.1016/j.mex.2021.101576)
Supplement: Supplementary file 1 [file mmc1.pdf]

		Forward (5'-3')	Reverse (5'-3')
16S	sample01	GAGCCTCTCATTGTCCGTTCTCTAAGAGTTTGATCMTGGCTCAG	TGCGTACAGCAATCAGTTACATTGGGTTACCTTGTTACGACTT
	sample02	GAGCCTCTCATTGTCCGTTCTCTAAGAGTTTGATCMTGGCTCAG	CCAGTAGAAGTCCGACAACGTCATGGTTACCTTGTTACGACTT
	sample03	GAGCCTCTCATTGTCCGTTCTCTAAGAGTTTGATCMTGGCTCAG	CAGACTTGGTACGGTTGGGTAACTGGTTACCTTGTTACGACTT
	sample04	GAGCCTCTCATTGTCCGTTCTCTAAGAGTTTGATCMTGGCTCAG	GGACGAAGAACTCAAGTCAAAGGCGGTTACCTTGTTACGACTT
	sample05	GAGCCTCTCATTGTCCGTTCTCTAAGAGTTTGATCMTGGCTCAG	CTACTTACGAAGCTGAGGGACTGCGGTTACCTTGTTACGACTT
	sample06	GAGCCTCTCATTGTCCGTTCTCTAAGAGTTTGATCMTGGCTCAG	ATGTCCCAGTTAGAGGAGGAAACAGGTTACCTTGTTACGACTT
	sample07	GAGCCTCTCATTGTCCGTTCTCTAAGAGTTTGATCMTGGCTCAG	GCTTGCGATTGATGCTTAGTATCAGGTTACCTTGTTACGACTT
	sample08	GAGCCTCTCATTGTCCGTTCTCTAAGAGTTTGATCMTGGCTCAG	ACCACAGGAGGACGATACAGAGAAGGTTACCTTGTTACGACTT
	sample09	ACCACTGCCATGTATCAAAGTACGAGAGTTTGATCMTGGCTCAG	TGCGTACAGCAATCAGTTACATTGGGTTACCTTGTTACGACTT
	sample10	ACCACTGCCATGTATCAAAGTACGAGAGTTTGATCMTGGCTCAG	CCAGTAGAAGTCCGACAACGTCATGGTTACCTTGTTACGACTT
	sample11	ACCACTGCCATGTATCAAAGTACGAGAGTTTGATCMTGGCTCAG	CAGACTTGGTACGGTTGGGTAACTGGTTACCTTGTTACGACTT
	sample12	ACCACTGCCATGTATCAAAGTACGAGAGTTTGATCMTGGCTCAG	GGACGAAGAACTCAAGTCAAAGGCGGTTACCTTGTTACGACTT
	sample13	ACCACTGCCATGTATCAAAGTACGAGAGTTTGATCMTGGCTCAG	CTACTTACGAAGCTGAGGGACTGCGGTTACCTTGTTACGACTT
	sample14	ACCACTGCCATGTATCAAAGTACGAGAGTTTGATCMTGGCTCAG	ATGTCCCAGTTAGAGGAGGAAACAGGTTACCTTGTTACGACTT
	sample15	ACCACTGCCATGTATCAAAGTACGAGAGTTTGATCMTGGCTCAG	GCTTGCGATTGATGCTTAGTATCAGGTTACCTTGTTACGACTT
	sample16	ACCACTGCCATGTATCAAAGTACGAGAGTTTGATCMTGGCTCAG	ACCACAGGAGGACGATACAGAGAAGGTTACCTTGTTACGACTT
	sample17	CTTACTACCCAGTGAACCTCCTCGAGAGTTTGATCMTGGCTCAG	TGCGTACAGCAATCAGTTACATTGGGTTACCTTGTTACGACTT
	sample18	CTTACTACCCAGTGAACCTCCTCGAGAGTTTGATCMTGGCTCAG	CCAGTAGAAGTCCGACAACGTCATGGTTACCTTGTTACGACTT
	sample19	CTTACTACCCAGTGAACCTCCTCGAGAGTTTGATCMTGGCTCAG	CAGACTTGGTACGGTTGGGTAACTGGTTACCTTGTTACGACTT
	sample20	CTTACTACCCAGTGAACCTCCTCGAGAGTTTGATCMTGGCTCAG	GGACGAAGAACTCAAGTCAAAGGCGGTTACCTTGTTACGACTT
	sample21	CTTACTACCCAGTGAACCTCCTCGAGAGTTTGATCMTGGCTCAG	CTACTTACGAAGCTGAGGGACTGCGGTTACCTTGTTACGACTT
	sample22	CTTACTACCCAGTGAACCTCCTCGAGAGTTTGATCMTGGCTCAG	ATGTCCCAGTTAGAGGAGGAAACAGGTTACCTTGTTACGACTT
	sample23	CTTACTACCCAGTGAACCTCCTCGAGAGTTTGATCMTGGCTCAG	GCTTGCGATTGATGCTTAGTATCAGGTTACCTTGTTACGACTT
	sample24	CTTACTACCCAGTGAACCTCCTCGAGAGTTTGATCMTGGCTCAG	ACCACAGGAGGACGATACAGAGAAGGTTACCTTGTTACGACTT
	sample25	GCATAGTTCTGCATGATGGGTTAGAGAGTTTGATCMTGGCTCAG	TGCGTACAGCAATCAGTTACATTGGGTTACCTTGTTACGACTT
	sample26	GCATAGTTCTGCATGATGGGTTAGAGAGTTTGATCMTGGCTCAG	CCAGTAGAAGTCCGACAACGTCATGGTTACCTTGTTACGACTT
	sample27	GCATAGTTCTGCATGATGGGTTAGAGAGTTTGATCMTGGCTCAG	CAGACTTGGTACGGTTGGGTAACTGGTTACCTTGTTACGACTT
	sample28	GCATAGTTCTGCATGATGGGTTAGAGAGTTTGATCMTGGCTCAG	GGACGAAGAACTCAAGTCAAAGGCGGTTACCTTGTTACGACTT
	sample29	GCATAGTTCTGCATGATGGGTTAGAGAGTTTGATCMTGGCTCAG	CTACTTACGAAGCTGAGGGACTGCGGTTACCTTGTTACGACTT
	sample30	GCATAGTTCTGCATGATGGGTTAGAGAGTTTGATCMTGGCTCAG	ATGTCCCAGTTAGAGGAGGAAACAGGTTACCTTGTTACGACTT
	sample31	GCATAGTTCTGCATGATGGGTTAGAGAGTTTGATCMTGGCTCAG	GCTTGCGATTGATGCTTAGTATCAGGTTACCTTGTTACGACTT
	sample32	GCATAGTTCTGCATGATGGGTTAGAGAGTTTGATCMTGGCTCAG	ACCACAGGAGGACGATACAGAGAAGGTTACCTTGTTACGACTT
	sample33	GTAAGTTGGGTATGCAACGCAATGAGAGTTTGATCMTGGCTCAG	TGCGTACAGCAATCAGTTACATTGGGTTACCTTGTTACGACTT
	sample34	GTAAGTTGGGTATGCAACGCAATGAGAGTTTGATCMTGGCTCAG	CCAGTAGAAGTCCGACAACGTCATGGTTACCTTGTTACGACTT
	sample35	GTAAGTTGGGTATGCAACGCAATGAGAGTTTGATCMTGGCTCAG	CAGACTTGGTACGGTTGGGTAACTGGTTACCTTGTTACGACTT
	sample36	GTAAGTTGGGTATGCAACGCAATGAGAGTTTGATCMTGGCTCAG	GGACGAAGAACTCAAGTCAAAGGCGGTTACCTTGTTACGACTT
	sample37	GTAAGTTGGGTATGCAACGCAATGAGAGTTTGATCMTGGCTCAG	CTACTTACGAAGCTGAGGGACTGCGGTTACCTTGTTACGACTT
	sample38	GTAAGTTGGGTATGCAACGCAATGAGAGTTTGATCMTGGCTCAG	ATGTCCCAGTTAGAGGAGGAAACAGGTTACCTTGTTACGACTT
ITS	sample39	AAGAAAGTTGTCGGTGTCTTTGTGTCCGTAGGTGAACCTGCGG	GTTTCATCTATCGGAGGGAATGGATCCTCCGCTTATTGATATGC
	sample40	AAGAAAGTTGTCGGTGTCTTTGTGTCCGTAGGTGAACCTGCGG	CAGGTAGAAAGAAGCAGAATCGGATCCTCCGCTTATTGATATGC
	sample41	AAGAAAGTTGTCGGTGTCTTTGTGTCCGTAGGTGAACCTGCGG	AGAACGACTTCCATACTCGTGTGATCCTCCGCTTATTGATATGC
	sample42	AAGAAAGTTGTCGGTGTCTTTGTGTCCGTAGGTGAACCTGCGG	AACGAGTCTCTTGGGACCCATAGATCCTCCGCTTATTGATATGC
	sample43	AAGAAAGTTGTCGGTGTCTTTGTGTCCGTAGGTGAACCTGCGG	AGGTCTACCTCGCTAACACCACTGTCCTCCGCTTATTGATATGC
	sample44	AAGAAAGTTGTCGGTGTCTTTGTGTCCGTAGGTGAACCTGCGG	CGTCAACTGACAGTGGTTCGTACTTCCTCCGCTTATTGATATGC
	sample45	AAGAAAGTTGTCGGTGTCTTTGTGTCCGTAGGTGAACCTGCGG	ACCCTCCAGGAAAGTACCTCTGATTCCTCCGCTTATTGATATGC
	sample46	AAGAAAGTTGTCGGTGTCTTTGTGTCCGTAGGTGAACCTGCGG	CCAAACCCAACAACCTAGATAGGCTCCTCCGCTTATTGATATGC
	sample47	TCGATTCCGTTTGTAGTCGTCTGTTCCGTAGGTGAACCTGCGG	GTTTCATCTATCGGAGGGAATGGATCCTCCGCTTATTGATATGC
	sample48	TCGATTCCGTTTGTAGTCGTCTGTTCCGTAGGTGAACCTGCGG	CAGGTAGAAAGAAGCAGAATCGGATCCTCCGCTTATTGATATGC
	sample49	TCGATTCCGTTTGTAGTCGTCTGTTCCGTAGGTGAACCTGCGG	AGAACGACTTCCATACTCGTGTGATCCTCCGCTTATTGATATGC
	sample50	TCGATTCCGTTTGTAGTCGTCTGTTCCGTAGGTGAACCTGCGG	AACGAGTCTCTTGGGACCCATAGATCCTCCGCTTATTGATATGC
	sample51	TCGATTCCGTTTGTAGTCGTCTGTTCCGTAGGTGAACCTGCGG	AGGTCTACCTCGCTAACACCACTGTCCTCCGCTTATTGATATGC
	sample52	TCGATTCCGTTTGTAGTCGTCTGTTCCGTAGGTGAACCTGCGG	CGTCAACTGACAGTGGTTCGTACTTCCTCCGCTTATTGATATGC

Appendix 1 Primer pairs used in PCR to obtain the 1st Amplicon

		Primer pair used for the first PCR		Primer pair used for the second PCR	
		Forward (5'-3')	Reverse (5'-3')	Forward (5'-3')	Reverse (5'-3')
16S	sample01	CATACAGCGACTACGCATTCTCATAGAGTTTGATCMTGGCTCAG	TGCGTACAGCAATCAGTTACATTGGGTTACCTTGTTACGACTT	CCGATCCTTGTGGCTTCTAACTTCGGCATACAGCGACTACGCATTCTCAT	CCTGGGAGCATCAGGTAGTAACAGGGTGCGTACAGCAATCAGTTACATTG
	sample02	CATACAGCGACTACGCATTCTCATAGAGTTTGATCMTGGCTCAG	CCAGTAGAAGTCCGACAACGTCATGGTTACCTTGTTACGACTT	CCGATCCTTGTGGCTTCTAACTTCGGCATACAGCGACTACGCATTCTCAT	TAGCTGACTGTCTTCCATACCGACGGCCAGTAGAAGTCCGACAACGTCAT
	sample03	CATACAGCGACTACGCATTCTCATAGAGTTTGATCMTGGCTCAG	CAGACTTGGTACGGTTGGGTAACTGGTTACCTTGTTACGACTT	CCGATCCTTGTGGCTTCTAACTTCGGCATACAGCGACTACGCATTCTCAT	AAGAAACAGGATGACAGAACCCTCGGCAGACTTGGTACGGTTGGGTAACT
	sample04	CATACAGCGACTACGCATTCTCATAGAGTTTGATCMTGGCTCAG	GGACGAAGAACTCAAGTCAAAGGCGGTTACCTTGTTACGACTT	CCGATCCTTGTGGCTTCTAACTTCGGCATACAGCGACTACGCATTCTCAT	TACAAGCATCCCAACACTTCCACTGGGGACGAAGAACTCAAGTCAAAGGC
	sample05	CATACAGCGACTACGCATTCTCATAGAGTTTGATCMTGGCTCAG	CTACTTACGAAGCTGAGGGACTGCGGTTACCTTGTTACGACTT	CCGATCCTTGTGGCTTCTAACTTCGGCATACAGCGACTACGCATTCTCAT	GACCATTGTGATGAACCCTGTTGTGGCTACTTACGAAGCTGAGGGACTGC
	sample06	CATACAGCGACTACGCATTCTCATAGAGTTTGATCMTGGCTCAG	ATGTCCCAGTTAGAGGAGGAAACAGGTTACCTTGTTACGACTT	CCGATCCTTGTGGCTTCTAACTTCGGCATACAGCGACTACGCATTCTCAT	ATGCTTGTTACATCAACCCTGGACGGATGTCCCAGTTAGAGGAGGAAACA
	sample07	CATACAGCGACTACGCATTCTCATAGAGTTTGATCMTGGCTCAG	GCTTGCGATTGATGCTTAGTATCAGGTTACCTTGTTACGACTT	CCGATCCTTGTGGCTTCTAACTTCGGCATACAGCGACTACGCATTCTCAT	CGACCTGTTTCTCAGGGATACAACGGGCTTGCGATTGATGCTTAGTATCA
	sample08	CATACAGCGACTACGCATTCTCATAGAGTTTGATCMTGGCTCAG	ACCACAGGAGGACGATACAGAGAAGGTTACCTTGTTACGACTT	CCGATCCTTGTGGCTTCTAACTTCGGCATACAGCGACTACGCATTCTCAT	AACAACCGAACCTTTGAATCAGAAGGACCACAGGAGGACGATACAGAGAA
	sample09	CGACGGTTAGATTCACCTCTTACAAGAGTTTGATCMTGGCTCAG	TGCGTACAGCAATCAGTTACATTGGGTTACCTTGTTACGACTT	GTTTGTCATACTCGTGTGCTCACCGGCGACGGTTAGATTCACCTCTTACA	CCTGGGAGCATCAGGTAGTAACAGGGTGCGTACAGCAATCAGTTACATTG
	sample10	CGACGGTTAGATTCACCTCTTACAAGAGTTTGATCMTGGCTCAG	CCAGTAGAAGTCCGACAACGTCATGGTTACCTTGTTACGACTT	GTTTGTCATACTCGTGTGCTCACCGGCGACGGTTAGATTCACCTCTTACA	TAGCTGACTGTCTTCCATACCGACGGCCAGTAGAAGTCCGACAACGTCAT
	sample11	CGACGGTTAGATTCACCTCTTACAAGAGTTTGATCMTGGCTCAG	CAGACTTGGTACGGTTGGGTAACTGGTTACCTTGTTACGACTT	GTTTGTCATACTCGTGTGCTCACCGGCGACGGTTAGATTCACCTCTTACA	AAGAAACAGGATGACAGAACCCTCGGCAGACTTGGTACGGTTGGGTAACT
	sample12	CGACGGTTAGATTCACCTCTTACAAGAGTTTGATCMTGGCTCAG	GGACGAAGAACTCAAGTCAAAGGCGGTTACCTTGTTACGACTT	GTTTGTCATACTCGTGTGCTCACCGGCGACGGTTAGATTCACCTCTTACA	TACAAGCATCCCAACACTTCCACTGGGGACGAAGAACTCAAGTCAAAGGC
	sample13	CGACGGTTAGATTCACCTCTTACAAGAGTTTGATCMTGGCTCAG	CTACTTACGAAGCTGAGGGACTGCGGTTACCTTGTTACGACTT	GTTTGTCATACTCGTGTGCTCACCGGCGACGGTTAGATTCACCTCTTACA	GACCATTGTGATGAACCCTGTTGTGGCTACTTACGAAGCTGAGGGACTGC
	sample14	CGACGGTTAGATTCACCTCTTACAAGAGTTTGATCMTGGCTCAG	ATGTCCCAGTTAGAGGAGGAAACAGGTTACCTTGTTACGACTT	GTTTGTCATACTCGTGTGCTCACCGGCGACGGTTAGATTCACCTCTTACA	ATGCTTGTTACATCAACCCTGGACGGATGTCCCAGTTAGAGGAGGAAACA
ITS	sample15	GAGTCTTGTGTCCCAGTTACCAGGTCCGTAGGTGAACCTGCGG	GTTTCATCTATCGGAGGGAATGGATCCTCCGCTTATTGATATGC	CTTTCGTTGTTGACTCGACGGTAGGGGAGTCTTGTGTCCCAGTTACCAGG	GTTGAATGAGCCTACTGGGTCCTCGGGTTTCATCTATCGGAGGGAATGGA
	sample16	GAGTCTTGTGTCCCAGTTACCAGGTCCGTAGGTGAACCTGCGG	CAGGTAGAAAGAAGCAGAATCGGATCCTCCGCTTATTGATATGC	CTTTCGTTGTTGACTCGACGGTAGGGGAGTCTTGTGTCCCAGTTACCAGG	TGAGAGACAAGATTGTTCGTGGACGGCAGGTAGAAAGAAGCAGAATCGGA
	sample17	GAGTCTTGTGTCCCAGTTACCAGGTCCGTAGGTGAACCTGCGG	AGAACGACTTCCATACTCGTGTGATCCTCCGCTTATTGATATGC	CTTTCGTTGTTGACTCGACGGTAGGGGAGTCTTGTGTCCCAGTTACCAGG	AGATTCAGACCGTCTCATGCAAAGGGAGAACGACTTCCATACTCGTGTGA
	sample18	GAGTCTTGTGTCCCAGTTACCAGGTCCGTAGGTGAACCTGCGG	AACGAGTCTCTTGGGACCCATAGATCCTCCGCTTATTGATATGC	CTTTCGTTGTTGACTCGACGGTAGGGGAGTCTTGTGTCCCAGTTACCAGG	CAAGAGCTTTGACTAAGGAGCATGGGAACGAGTCTCTTGGGACCCATAGA
	sample19	GAGTCTTGTGTCCCAGTTACCAGGTCCGTAGGTGAACCTGCGG	AGGTCTACCTCGCTAACACCACTGTCCTCCGCTTATTGATATGC	CTTTCGTTGTTGACTCGACGGTAGGGGAGTCTTGTGTCCCAGTTACCAGG	TGGAAGATGAGACCCTGATCTACGGGAGGTCTACCTCGCTAACACCACTG
	sample20	GAGTCTTGTGTCCCAGTTACCAGGTCCGTAGGTGAACCTGCGG	CGTCAACTGACAGTGGTTCGTACTTCCTCCGCTTATTGATATGC	CTTTCGTTGTTGACTCGACGGTAGGGGAGTCTTGTGTCCCAGTTACCAGG	TCACTACTCAACAGGTGGCATGAAGGCGTCAACTGACAGTGGTTCGTACT
	sample21	GAGTCTTGTGTCCCAGTTACCAGGTCCGTAGGTGAACCTGCGG	ACCCTCCAGGAAAGTACCTCTGATTCCTCCGCTTATTGATATGC	CTTTCGTTGTTGACTCGACGGTAGGGGAGTCTTGTGTCCCAGTTACCAGG	GCTAGGTCAATCTCCTTCGGAAGTGGACCCTCCAGGAAAGTACCTCTGAT
	sample22	GAGTCTTGTGTCCCAGTTACCAGGTCCGTAGGTGAACCTGCGG	CCAAACCCAACAACCTAGATAGGCTCCTCCGCTTATTGATATGC	CTTTCGTTGTTGACTCGACGGTAGGGGAGTCTTGTGTCCCAGTTACCAGG	CAGGTTACTCCTCCGTGAGTCTGAGGCCAAACCCAACAACCTAGATAGGC
	sample23	TTCGGATTCTATCGTGTTTCCCTATCCGTAGGTGAACCTGCGG	GTTTCATCTATCGGAGGGAATGGATCCTCCGCTTATTGATATGC	AGTAGAAAGGGTTCCTTCCCACTCGGTTCGGATTCTATCGTGTTTCCCTA	GTTGAATGAGCCTACTGGGTCCTCGGGTTTCATCTATCGGAGGGAATGGA
	sample24	TTCGGATTCTATCGTGTTTCCCTATCCGTAGGTGAACCTGCGG	CAGGTAGAAAGAAGCAGAATCGGATCCTCCGCTTATTGATATGC	AGTAGAAAGGGTTCCTTCCCACTCGGTTCGGATTCTATCGTGTTTCCCTA	TGAGAGACAAGATTGTTCGTGGACGGCAGGTAGAAAGAAGCAGAATCGGA
	sample25	TTCGGATTCTATCGTGTTTCCCTATCCGTAGGTGAACCTGCGG	AGAACGACTTCCATACTCGTGTGATCCTCCGCTTATTGATATGC	AGTAGAAAGGGTTCCTTCCCACTCGGTTCGGATTCTATCGTGTTTCCCTA	AGATTCAGACCGTCTCATGCAAAGGGAGAACGACTTCCATACTCGTGTGA
	sample26	TTCGGATTCTATCGTGTTTCCCTATCCGTAGGTGAACCTGCGG	AACGAGTCTCTTGGGACCCATAGATCCTCCGCTTATTGATATGC	AGTAGAAAGGGTTCCTTCCCACTCGGTTCGGATTCTATCGTGTTTCCCTA	CAAGAGCTTTGACTAAGGAGCATGGGAACGAGTCTCTTGGGACCCATAGA
	sample27	TTCGGATTCTATCGTGTTTCCCTATCCGTAGGTGAACCTGCGG	AGGTCTACCTCGCTAACACCACTGTCCTCCGCTTATTGATATGC	AGTAGAAAGGGTTCCTTCCCACTCGGTTCGGATTCTATCGTGTTTCCCTA	TGGAAGATGAGACCCTGATCTACGGGAGGTCTACCTCGCTAACACCACTG
	sample28	TTCGGATTCTATCGTGTTTCCCTATCCGTAGGTGAACCTGCGG	CGTCAACTGACAGTGGTTCGTACTTCCTCCGCTTATTGATATGC	AGTAGAAAGGGTTCCTTCCCACTCGGTTCGGATTCTATCGTGTTTCCCTA	TCACTACTCAACAGGTGGCATGAAGGCGTCAACTGACAGTGGTTCGTACT

Appendix 2 Primer pairs used in PCR to obtain the 2nd Amplicon

Sample	DNA concentration (ng/µl)
A:I	69.8
A:II	22.6
A:III	72.2
A:IV-1	0.3
A:IV-2	64.6
A:V-1	0.0107
A:V-2	37.4
A:VI-1	0.322
A:VI-2	52.8
A:1	0.0388
A:2	0.0256
A:3	0.0055
A:4-1	44.4
A:4-2	0.0282
A:5-1	0.028
A:5-2	0
A:6-1	54.6
A:6-2	0.274
B:I	43.2
B:II	34.6
B:III	23.4
B:IV-1	13.7
B:IV-2	28
B:V-1	9.88
B:V-2	34
B:VI-1	19.4
B:VI-2	60.8
B:1	9.98
B:2	11.6
B:3	7.3
B:4	21.4
B:5	6.6
B:6	22.6
B:7	1.92
B:8	68.4
B:9	21.8
leaf-r	0.884
leaf-s	0.824

Appendix 3 Concentration of DNA extracted from the samples

A and B represent samples at the beginning of the experiment and after 2 weeks, respectively.

Roman numerals and arithmetic numerals represent the samples of raw and sterile soil, respectively.

Leaf represents the fallen leaves of *Euptelea*.

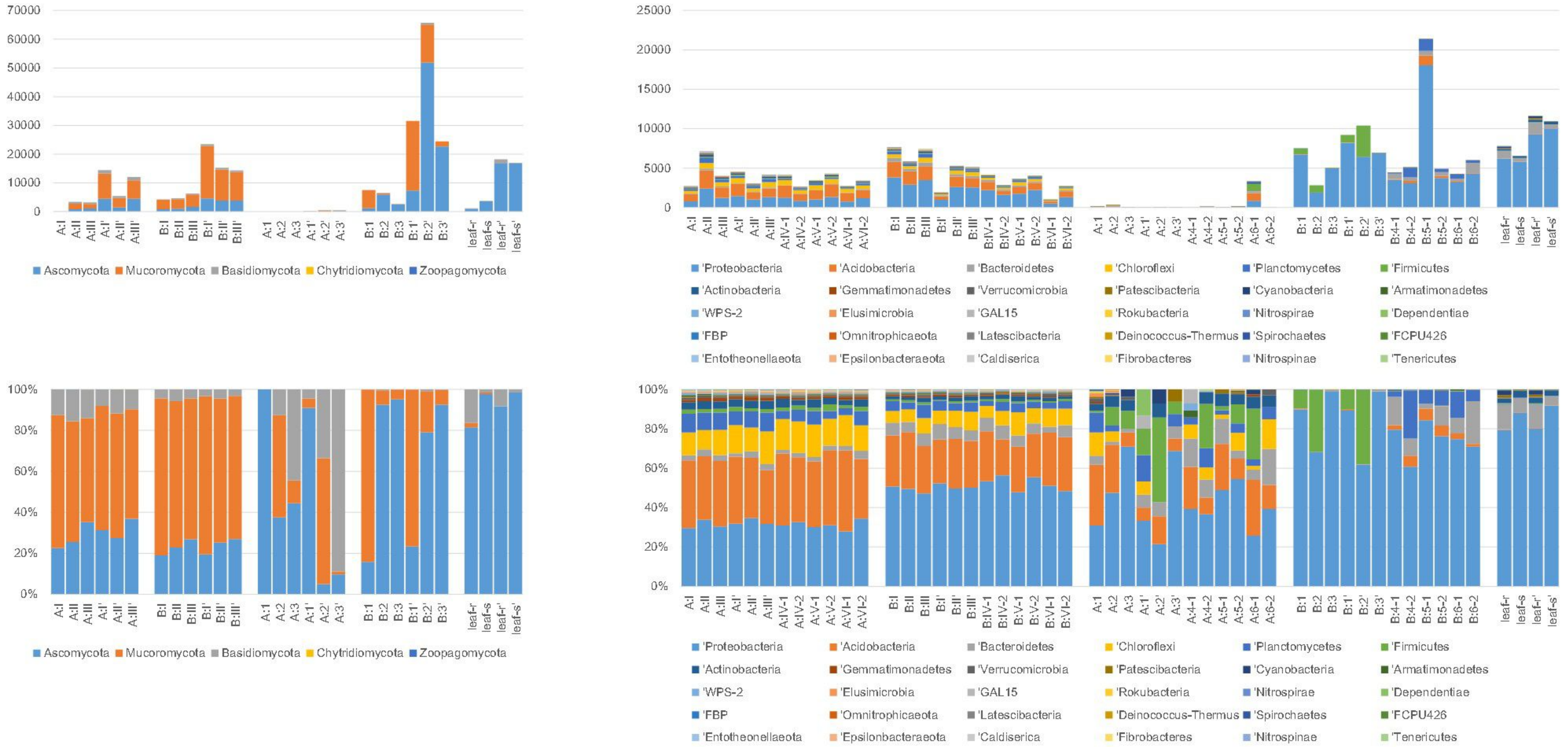

Appendix 4 The composition of phylum-level fungi obtained by analysis of the ITS region (left) and the composition of phylum-level bacteria obtained by analysis of the 16S region (right).
The top panel indicates the total of Ns in Tables 1 to 3, and the bottom panel presents the appearance ratio.
The horizontal axis represents the sample, and the abbreviations are as follows.
A and B represent samples at the beginning of the experiment and after 2 weeks, respectively.
Roman numerals and arithmetic numerals represent the raw and sterile soil samples, respectively.
Leaf represents the fallen leaves of *Euptelea.*
Indicators without apostrophes at the end represent the 1st Amplicon and 2nd Amplicon.

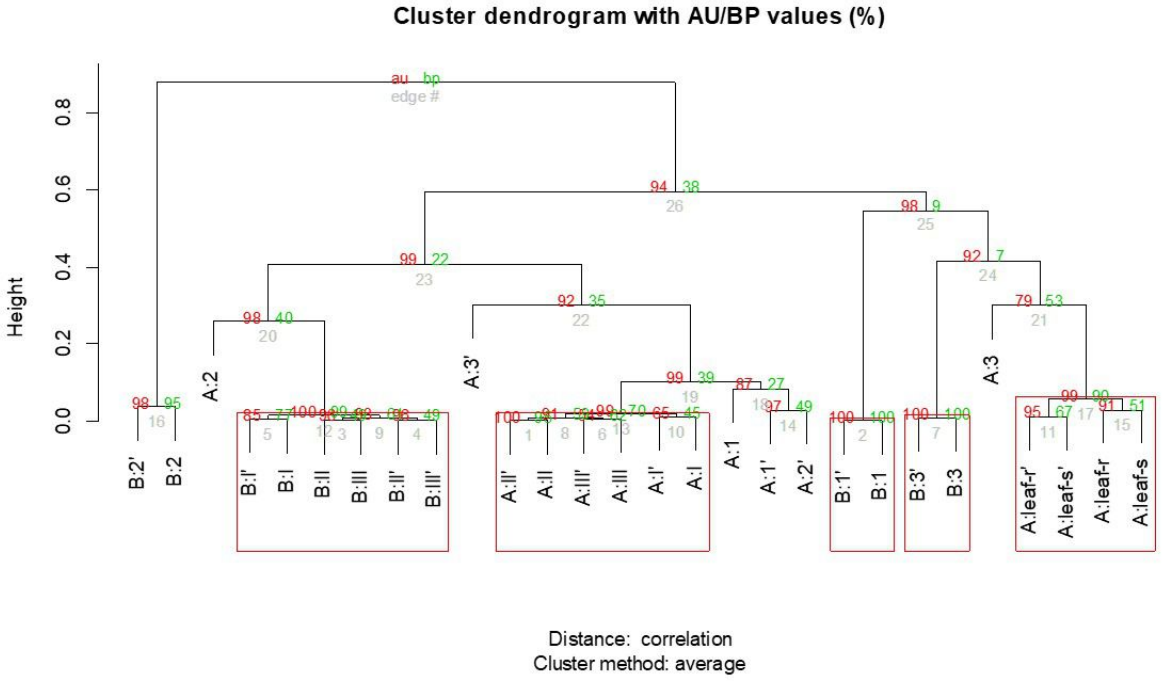

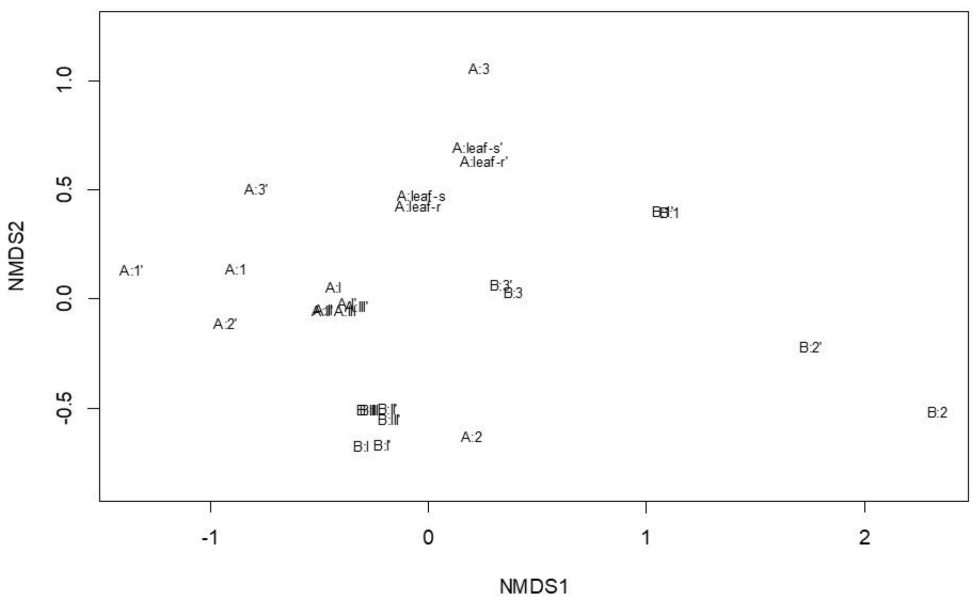

Appendix 5 Results of clustering analysis (left) and NMDS (right) of the 1st Amplicon and 2nd Amplicon in the ITS region
A and B represent the samples at the beginning of the experiment and after 2 weeks, respectively.
The Roman numerals and arithmetic numerals represent samples of raw and sterile soil, respectively.
Leaf represents the fallen leaves of *Euptelea*.
Indicators without apostrophes at the end represent the 1st Amplicon and 2nd Amplicon.
The red frame shows clusters with an α value of 0.99 or more.

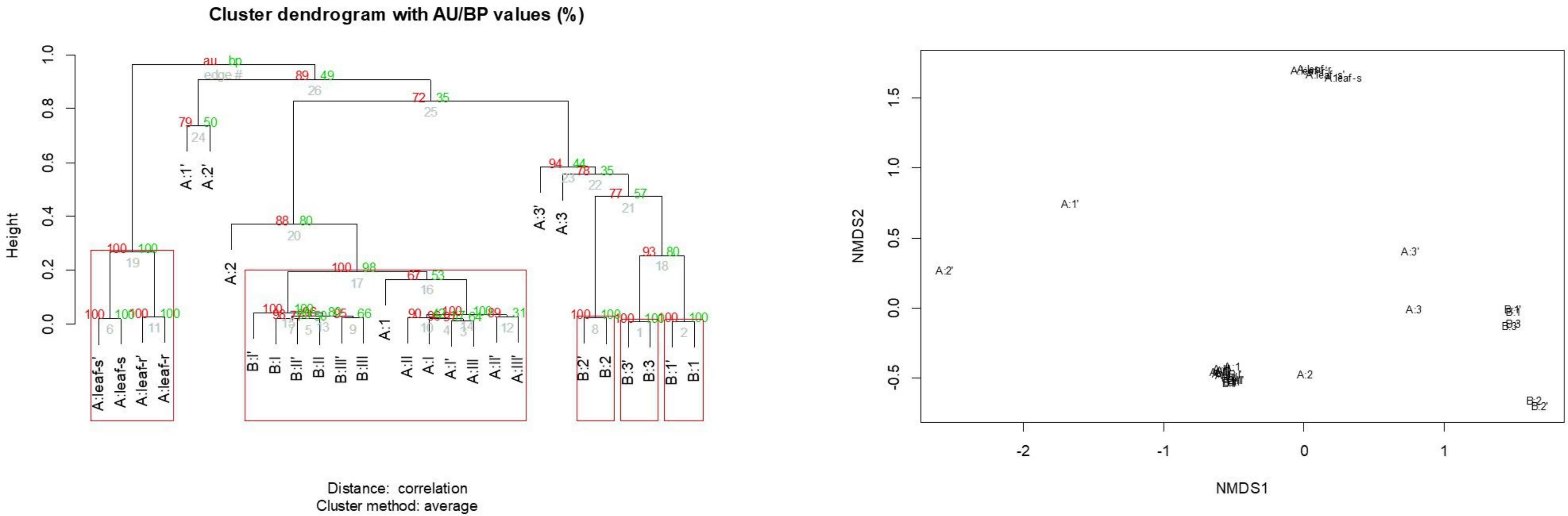

Appendix 6 Results of clustering analysis (left) and NMDS (right) of the 1st Amplicon and 2nd Amplicon in the 16S region.
A and B represent the samples at the beginning of the experiment and after 2 weeks, respectively.
The Roman numerals and arithmetic numerals represent samples of raw and sterile soil, respectively.
Leaf represents the fallen leaves of *Euptelea*.
Indicators without apostrophes at the end represent the 1st Amplicon and 2nd Amplicon.
The red frame shows clusters with an α value of 0.99 or more.

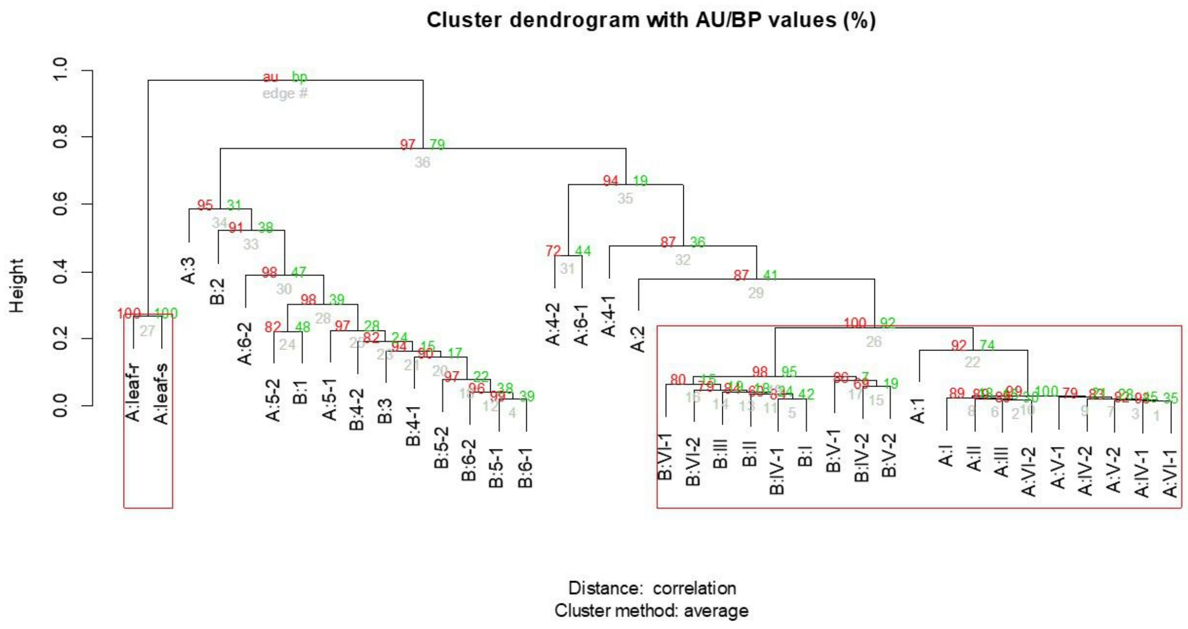

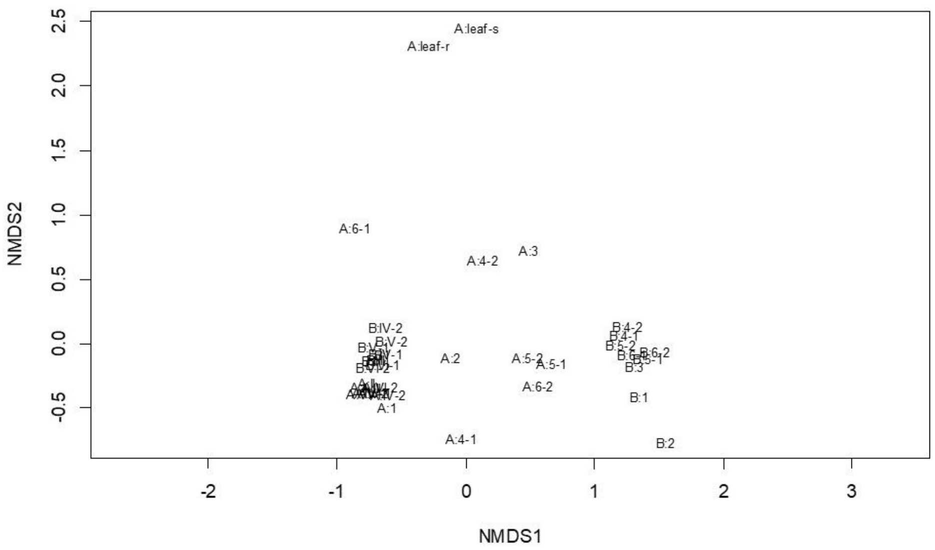

Appendix 7 Results of the 1st Amplicon clustering analysis (top) and NMDS (bottom) in the 16S region
A and B represent the samples at the beginning of the experiment and after 2 weeks, respectively.
I-VI-2 and 1-6-2 represent the samples of raw and sterile soil, respectively.
Leaf represents the fallen leaves of *Euptelea*.
The red frame shows clusters with an α value of 0.99 or more.
